# Endpoint Quaking-Induced Conversion: a Sensitive, Specific, and High-Throughput Method for Antemortem Diagnosis of Creutzfeldt-Jacob Disease

**DOI:** 10.1128/JCM.00542-16

**Published:** 2016-06-24

**Authors:** Keding Cheng, Robert Vendramelli, Angela Sloan, Brooks Waitt, Lisa Podhorodecki, Debra Godal, J. David Knox

**Affiliations:** a National Microbiology Laboratory, Public Health Agency of Canada, Winnipeg, Manitoba, Canada; b Department of Human Anatomy and Cell Sciences, Faculty of Medicine, University of Manitoba, Winnipeg, Manitoba, Canada; c Department of Medical Microbiology, Faculty of Medicine, University of Manitoba, Winnipeg, Manitoba, Canada; Rhode Island Hospital

## Abstract

The Prion Laboratory Section of the Public Health Agency of Canada supports heath care professionals dealing with patients suspected to have Creutzfeldt-Jakob disease (CJD) by testing cerebrospinal fluid (CSF) for protein markers of CJD. To better serve Canadian diagnostic requirements, a quaking-induced conversion (QuIC)-based assay has been added to the test panel. The QuIC tests exploit the ability of disease-associated prion protein, found in the CSF of a majority of CJD patients, to convert a recombinant prion protein (rPrP) into detectable amounts of a misfolded, aggregated form of rPrP. The rPrP aggregates interact with a specific dye, causing a measurable change in the dye's fluorescence emission spectrum. Optimal test and analysis parameters were empirically determined. Taking both practical and performance considerations into account, an endpoint QuIC (EP-QuIC) configuration was chosen. EP-QuIC uses a thermo-mixer to perform the shaking necessary to produce the quaking-induced conversions. Fluorescence readings are obtained from a microwell fluorescence reader only at the beginning and the end of EP-QuIC reactions. Samples for which the relative fluorescence unit ratio between the initial and final readings represent a ≥4 increase in signal intensity in at least two of the three replicates are classified as positive. A retrospective analysis of 91 CSF samples that included 45 confirmed cases of CJD and 46 non-CJD cases was used to estimate the performance characteristics of the EP-QuIC assay. The diagnostic sensitivity and specificity of the EP-QuIC test of this set of samples were 98 and 91%, respectively.

## INTRODUCTION

Subacute encephalopathies, including Creutzfeldt-Jakob disease (CJD), constitute a large and heterogeneous group of brain diseases that can be diagnostically challenging (1). Among these diseases, CJD has a special urgency, since this disease can be transmitted between individuals under some circumstances by an infection-like mechanism. Additional diagnostic difficulties arise due to the fact that the infectious agents are not conventional viruses or bacteria but rather are composed of a disease-associated, misfolded isoform (PrPd) of the host prion protein (PrPc) (2). This key biological fact precludes the use of conventional technologies, such as PCR and serology, that are commonly applied to the direct detection of other infectious agents.

The diagnostic approach for the most common form of CJD, sporadic CJD, has to date relied upon a standardized clinical presentation and supporting investigations such as the presence of increased amounts of indirect protein markers of disease found in patients' cerebrospinal fluid (CSF) (3, 4). The most commonly used markers are 14-3-3 protein (5), microtubule-associated protein tau (6), and S100 protein (7). These surrogate markers are not fully diagnostic due to the fact that they have variable sensitivities and are not specific for any form of CJD (8–10).

The advent of new tests that exploit the natural ability of PrPd to induce the conversion PrPc into a misfolded form *in vivo* represent a major advance in the diagnoses of sporadic CJD. In quaking-induced conversion (QuIC) (11) or real-time QuIC (RT-QuIC) assays (12), the prion-containing samples, such as brain homogenate or CSF, are added to wells containing recombinant PrP (rPrP). Cycles of shaking cause PrPd in the samples to convert the rPrP into a misfolded form. The accumulation of the resulting insoluble rPrP aggregates generated by this process then bind a fluorescent dye (thioflavin T), causing a change in the dye's fluorescence emission spectrum that can be measured in real time with a spectrofluorometer.

The high degree of sensitivity and specificity exhibited by RT-QuIC based tests in a growing number of laboratories strongly supports the inclusion of such a test in the clinical investigation of suspected cases of sporadic CJD and genetic CJD (13–18). In order to include a QuIC-based test in the reference services provided by the Public Health Agency of Canada's Prion Laboratory Section, a standardized protocol had to be established and the performance characteristics in the Canadian setting defined. This report describes in detail the QuIC test implemented at the Prion Laboratory Section and the results of retrospective analyses of 91 clinically defined CSF samples. QuIC with endpoint readings only, named EP-QuIC, was explored in parallel with RT-QuIC analyses (12–18).

## MATERIALS AND METHODS

This project, involving the use of human specimens of the CJD biobank housed at the National Microbiology Laboratory, was reviewed and approved by the Health Canada and Public Health Agency of Canada's Research Ethics Board (HC-PHAC REB 2014-0033). In total 91 CSF specimens, sent to the Prion Laboratory Section for testing due to suspicion of CJD, were used: 45 pathologically confirmed cases of CJD (43 for sporadic CJD and 2 for genetic CJD) and 46 clinically confirmed non-CJD cases. The final diagnoses of all samples are provided in Table S1 in the supplemental material. All sample handling was performed in a biological safety cabinet inside a biosafety level 3 containment laboratory.

The substrate used was Syrian hamster recombinant prion protein (rPrP, residues 23 to 231), provided by the Bristol Institute for Transfusion Sciences. Previously confirmed negative and positive CSF samples were run on each plate as controls. CSF analyses were performed in triplicate using black 96-well optical-bottom plates with 100-μl reaction volumes. Each well had 15 μl of CSF added to 85 μl of QuIC reaction buffer, resulting in a final composition of 300 mM NaCl, 10 μM EDTA, 10 μM ThT, and 0.1 mg/ml Syrian hamster rPrP in 10 mM phosphate buffer (pH 5.8). All the reagents were made fresh with mass spectrometry-grade water and passed through a 0.22-μm-pore size filter after preparation.

Sealing tape was applied and both RT-QuIC and EP-QuIC reaction mixtures were incubated at 42°C with intermittent shaking cycles consisting of 90 s of shaking at 900 rpm and 30 s of rest. Fluorescence readings were taken with an excitation wavelength of 450 nm and emission wavelength of 480 nm by a FLUOstar Omega plate reader. In RT-QuIC reactions the plate remained in the FLUOstar Omega plate reader with continued cycles of double orbital shaking and relative fluorescent unit (RFU) readings every 45 min for the duration of the 90-h experiment. In EP-QuIC, the plates were placed in an Eppendorf Thermomixer C equipped with a Smart Block plate and ThermoTop with shaking cycles consisting of 90 s of circular shaking at 900 rpm and 30 s of rest at 42°C. After 20 min, the plates were removed from the Eppendorf Thermomixer C, and an initial RFU reading was taken on a preheated FLUOstar Omega plate reader using the same excitation and emission spectra used for the RT-QuIC reactions (excitation wavelength, 450 nm; emission wavelength, 480 nm). The plates were then returned to the Eppendorf Thermomixer C, and the intermittent shaking cycles continued for 90 h. An endpoint reading was then taken on the FLOUstar Omega using the excitation and emission spectra described above.

At the end of the experiment, the RFU final/RFU initial ratio of each well was calculated. Based on receiver operating characteristic curve analyses, individual wells were scored as positive in RT-QuIC reactions when the RFU final/RFU initial was ≥2.0 (see Fig. S1 in the supplemental material). Positive EP-QuIC wells were determined to have a RFU final/RFU initial ratio of ≥4.0 (see Fig. S2 in the supplemental material). A sample was considered positive when at least two of the three assay replicates for a sample were positive. In instances where one of the three assay replicates for a sample was positive, the sample was reanalyzed using three different volumes of CSF in the reaction mixture (7.5, 15, and 30 μl). Samples consistently exhibiting one of the three assay replicate wells as positive were scored as indeterminate. The detailed method is described in in the supplemental material.

## RESULTS

**FIG 1:**
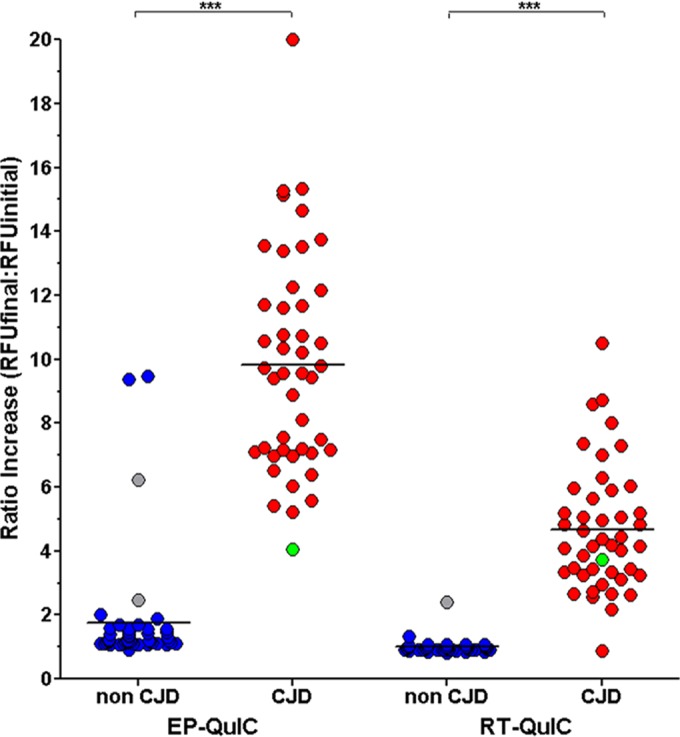
Distribution of CSF results (ratios) as determined by either EP-QuIC or RT-QuIC. Individual CSF results from non-CJD samples are colored blue (with indeterminate results in gray), whereas results from positive CJD samples are colored red (with indeterminate results in green). ***, *P* < 0.0001.

In the development of any diagnostic test a balance must be struck between its sensitivity and specificity. Too much emphasis on sensitivity increases the odds of false positives. Too much emphasis on specificity increases the odds of false negatives. In this respect EP-QuIC and RT-QuIC were slightly different. As shown in Fig. 1, EP-QuIC generated two false positives and RT-QuIC one false negative. There were also samples classified as indeterminate, seven resulting from the RT-QuIC method and four resulting from the EP-QuIC method. When these indeterminate samples were reanalyzed one of the four EP-QuIC indeterminate samples converted to a true negative and three remained indeterminate (one CJD and two non-CJD). Upon reanalysis of the seven RT-QuIC indeterminate samples, five were correctly classified, and two samples remained indeterminate (one CJD and one non-CJD). No clear correlation between the indeterminate or falsely classified samples and particular clinical diagnoses was observed (see Table S2 in the supplemental material). The results suggest that EP-QuIC was slightly more sensitive, whereas RT-QuIC was slightly more specific.

The reliability of the assay was assessed by having 8 CSF samples tested by two analysts on the same day, as well as the reanalysis of 41 samples by EP-QuIC on two separate dates. Both experiments demonstrated 100% agreement between all samples.

The results in Fig. 1 illustrate that the mean fold increase of the final RFU/initial RFU is greater in EP-QuIC than that for RT-QuIC. The increased responsiveness of the EP-QuIC reactions is also reflected in the optimal RFU ratio ranges calculated to maximize the sensitivity and specificity. Receiver operating characteristic curve analysis (see Fig. S1 and S2 in the supplemental material) indicated that the optimal RFU ratio range to maximize the sensitivity and specificity of RT-QuIC was between 1.755 and 2.295, whereas the optimal RFU ratio range for EP-QuIC was between 3.22 and 4.63. Since all the reagents are the same, it is suspected that the differences observed between EP-QuIC and RT-QuIC are due to the different modes of shaking used by the Eppendorf Thermomixer and the FLUOstar Omega. The FLUOstar Omega uses double orbital shaking, whereas the Eppendorf Thermomixer employs circular shaking.

Indeterminate samples did not return a defined positive or negative result and are therefore excluded from being considered true positive or true negative results. Instead the indeterminate samples known to be CJD positive are included in the false negatives and indeterminate samples known to be non-CJD are included in false positives in the calculation of sensitivity and specificity, as shown in Table 1. The inclusion of the 14-3-3 data, a well-known surrogate marker for sCJD (19), in Table 1 highlights the comparatively high levels of sensitivity and specificity achieved by both EP-QuIC and RT-QuIC (3, 19). Kappa statistics, used to determine the interobserver agreement (20), demonstrated that the two methods were in almost perfect agreement (see Table S3 in the supplemental material). Due to the differences in the prevalence of CJD positive CSF samples in the study population compared to that of samples sent to the Canadian prion laboratory (9), ongoing prospective analyses will be used to validate test performance characteristics in the clinical setting.

**TABLE 1:** Comparison of EP-QuIC, RT-QuIC, and 14-3-3 tests^*a*^

Test	No. of samples^*b*^						% Specificity or sensitivity (range)	
	CJD			Non-CJD			Sensitivity [A/(A+B+C)]	Specificity [D/(D+E+F)]
	TP (A)	FN (B)	ID (C)	TN (D)	FP (E)	ID (F)		
EP-QuIC	44	0	1	42	2	2	98 (88–100)	91 (79–98)
RT-QuIC	43	1	1	45	0	1	96 (85–99)	98 (88–100)
14-3-3	38	7	NA	32	14	NA	84 (71–94)	70 (54–82)

a 14-3-3 results are collective results obtained before EP-QuIC and RT-QuIC tests based on an ISO-certified method (9).

b Results are based on the final clinical diagnosis. TP, true positive; FN, false negative; ID, indeterminate; TN, true negative; FP, false positive; NA, not applicable.

## DISCUSSION

It had been previously determined by an EU Joint Programme–Neurodegenerative Disease Research (JPND) CSF QUIC consortium that the optimal shaking cycles for RT-QuIC reactions consisted of 90 s of shaking at 900 rpm, followed by a 30-s rest (21). The high rotation speed required for optimal performance exceeds the 700 rpm factory-set top speed of the FLUOstar Omega plate reader. A software package is available from BMG LabTech that will enable shaking at 900 rpm; however, installation and execution of this software results in the cancellation of the warranty. The increased shaking speed for extended periods of time increases the wear on the machine and ultimately leads to equipment failures. The resulting unscheduled interruption of QuIC tests cannot be tolerated in a clinical setting. The interruption of work flow can be mitigated by having multiple readers and rotating them through the level 3 biocontainment laboratory on an as needed basis. A more cost-effective and less cumbersome option is EP-QuIC, where the shaking is performed using an instrument such as the Eppendorf Thermomixer C equipped with a Smart Block plate and ThermoTop. Not only are these less expensive instruments, the much smaller footprint of the Eppendorf Thermomixer is also an important consideration given the limited bench space available in a level 3 biocontainment laboratory.

Time is obviously another important factor to consider when using such assays in a clinical setting. EP-QuIC throughput could be greatly increased by having multiple thermomixers and a single plate reader. Nonetheless, the 90-h run time discussed is relatively long in comparison to the enzyme-linked immunosorbent assays used to measure 14-3-3 and other surrogate markers of CJD in CSF. Normally, the Prion Laboratory Section runs samples received during the preceding week on Monday and Tuesday, with reports being issued on Wednesday. In order to ensure adherence to this schedule expected by referring physicians, the EP-QuIC reactions are started on Friday and read on Tuesday. Other laboratories are exploring the use of different substrates and run parameters that are reported to reduce the RT-QuIC run time to hours as opposed to days (22). These alternative protocols require further validation and need to become established in a number of laboratories before they can be considered suitable for the analysis of clinical specimens.

The lower cost and potentially higher throughput, coupled with sensitivity and specificity comparable to that of established RT-QuIC protocols, strongly support the inclusion of the EP-QuIC protocol described in the clinical investigation of suspected cases of CJD.

## Supplementary Material

Supplemental material
