# Supplementary material for: Endpoint Quaking-Induced Conversion: a Sensitive, Specific, and High-Throughput Method for Antemortem Diagnosis of Creutzfeldt-Jacob Disease
Source: J Clin Microbiol. 2016 Jun 24;54(7):1751–4. doi: 10.1128/JCM.00542-16 (PMC4922112; doi:10.1128/JCM.00542-16)
Supplement: Supplemental material [file supp_54_7_1751__index.html]

Supplemental material 

# Endpoint Quaking-Induced Conversion: a Sensitive, Specific, and High-Throughput Method for Antemortem Diagnosis of Creutzfeldt-Jacob Disease

## Supplemental material

- Supplemental file 1 -

  Supplemental methods (Detailed EP-QuIC procedure)

  PDF, 145K
- Supplemental file 2 -

  Fig. S1 (Receiver operating characteristic curve analysis for RT-QuIC results) and S2 (Receiver operating characteristic curve analysis for EP-QuIC) and Tables S1 (Clinical classification of samples), S2 (Clinical diagnoses of false-positive, false-negative, and intermediate samples), and S3 (Interobserver agreement analysis)

  PDF, 413K
