# Supplementary material for: Endpoint Quaking-Induced Conversion: a Sensitive, Specific, and High-Throughput Method for Antemortem Diagnosis of Creutzfeldt-Jacob Disease
Source: J Clin Microbiol. 2016 Jun 24;54(7):1751–4. doi: 10.1128/JCM.00542-16 (PMC4922112; doi:10.1128/JCM.00542-16)
Supplement: Supplemental material [file JCM.00542-16_zjm999095015so1.pdf]

Supplementary Method: Detailed EP-QuIC procedure

Materials:

Equipment and Supplies:
- Biological safety cabinet (BSC)
- Eppendorf Thermomixer C (Eppendorf, Catalogue #5382000023)
- Eppendorf ThermoTop (Eppendorf, Catalogue #5308000003)
- Eppendorf SmartBlock Plate (Eppendorf, Catalogue #5306000006)
- Fluorescence microplate reader with excitation filter at 450 nm and emission filter at 480 nm (e.g. BMG Labtech, FLUOstar Omega)
- 96-well plate with clear bottom, black (Thermo Fisher, Catalogue #265300)
- SealPlate adhesive microplate seal (E&K Scientific, Catalogue #T396100)

Reagents:
- Positive control: CSF obtained from a patient with prion disease
- Negative control: pooled CSF that has been confirmed not to induce the conversion of rPrP to rPrPd during EP-QuIC (Bioreclamation, Catalogue #HMCSF)
- Syrian hamster recombinant prion protein (rPrP), residues 23-231 (Bristol Institute for Transfusion Sciences)
- EDTA, 0.5 M, pH 8.0; (Fisher, Catalogue #BP2482-100)
- NaCl (Fisher, Catalogue #BP358)
- PBS, pH 7.4 (Sigma, Catalogue #P3813)
- Phosphate buffer, 10 mM, pH 5.8; prepared by adding 1.097 g of NaH_2PO_4 (Sigma, Catalogue #BP329) and 0.113 g Na_2HPO_4 (Sigma, Catalogue #S374) to 1 L of Optima LC/MS water
- Thioflavin T (Sigma, Catalogue #T3516-25G)
- Water, Optima LC/MS (Fisher, Catalogue #CAS7732-18-5)

Instructions:

1. Remove CSF samples from their storage location and place inside a BSC to thaw.

2. Turn on the thermomixer and pre-heat the unit by setting the incubation temperature to 42°C.

3. Prepare EP-QuIC Buffer (EPBQ) in 10 mM phosphate buffer, with final reagent concentrations of 138 mM PBS (pH 7.4), 162 mM NaCl, 10 µM EDTA, 10 µM Thioflavin T, and 0.1 mg/ml rPrP per well. Note: All reagent stocks should be diluted in Optima LC/MS water and filtered using a 0.22 µm membrane before addition to the EPQB. Vortex at low speed to mix.

4. Working in a BSC, load 85 µl of EPQB into the designated wells of a 96-well plate. Vortex controls and test samples for 10 seconds and load 15 µl into the designated wells of the plate. Note: CSF controls and samples should be run in triplicate.

5. Cover the plate with an adhesive microplate seal and place in the thermomixer. Replace the lid and run the following program:

Step	Temperature	Shaking Parameters	Time
Intermittent Shaking	42°C	900 rpm; 90 seconds shaking, 30 seconds rest	20 minutes
Storage	42°C	none	∞

6. Transfer the plate to a fluorescence microplate reader and take initial fluorescence readings at 450 nm excitation and 480 nm emission.

7. After the initial read, transfer the EP-QuIC plate back to the thermomixer and run the following program:

Step	Temperature	Shaking Parameters	Time
Intermittent Shaking	42°C	900 rpm; 90 seconds shaking, 30 seconds rest	90 hours
Storage	42°C	None	∞

8. Once the EP-QuIC run is complete, place the plate in the same fluorescence microplate reader and take final fluorescence readings with the same read parameters (excitation and emission filter, and gain setting) applied during the initial read.

9. Calculate individual fold increases for each control and test sample replicate by dividing the final RFU reading by the initial RFU reading. Calculate the average fold increase for each set of triplicates.

10. The individual fold increases of the positive control should be ≥ 4.0, while those of the negative control should be < 4.0. If the controls give the expected results, the test samples can be reported.

11. Test samples are reported as positive or negative using the individual and average fold increases:

    a. A sample is reported as positive if the individual fold increases of at least 2 of the replicates are ≥ 4.0 and the average fold increase is ≥ 4.0.

    b. A sample is reported as negative if the individual fold increases of all 3 replicates are < 4.0 and the average fold increase is < 4.0.

c. If the test does not meet the criteria listed above, the sample must be repeated using replicates of additional sample volumes.

    i. The test sample is retested using three sample volumes, each in triplicate. Volumes of 7.5 µl, 15 µl, and 30 µl of CSF are run.

    ii. A test sample is reported as positive if in any one set of volume replicates, the individual fold increases of at least 2 replicates are ≥ 4.0 and the average fold increase is ≥ 4.0.

    iii. A test sample is reported as negative if the individual fold increases of all 3 sets of volume replicates are < 4.0, and the average fold increase for all CSF volumes is < 4.0.

d. In rare cases, a test sample does not fall within the listed criteria after repeat testing. In these instances, the sample is reported as indeterminate.
