# Supplementary material for: Endpoint Quaking-Induced Conversion: a Sensitive, Specific, and High-Throughput Method for Antemortem Diagnosis of Creutzfeldt-Jacob Disease
Source: J Clin Microbiol. 2016 Jun 24;54(7):1751–4. doi: 10.1128/JCM.00542-16 (PMC4922112; doi:10.1128/JCM.00542-16)
Supplement: Supplemental material [file JCM.00542-16_zjm999095015so2.pdf]

Supplementary Figure 1. ROC curve analysis for RT-QuIC results

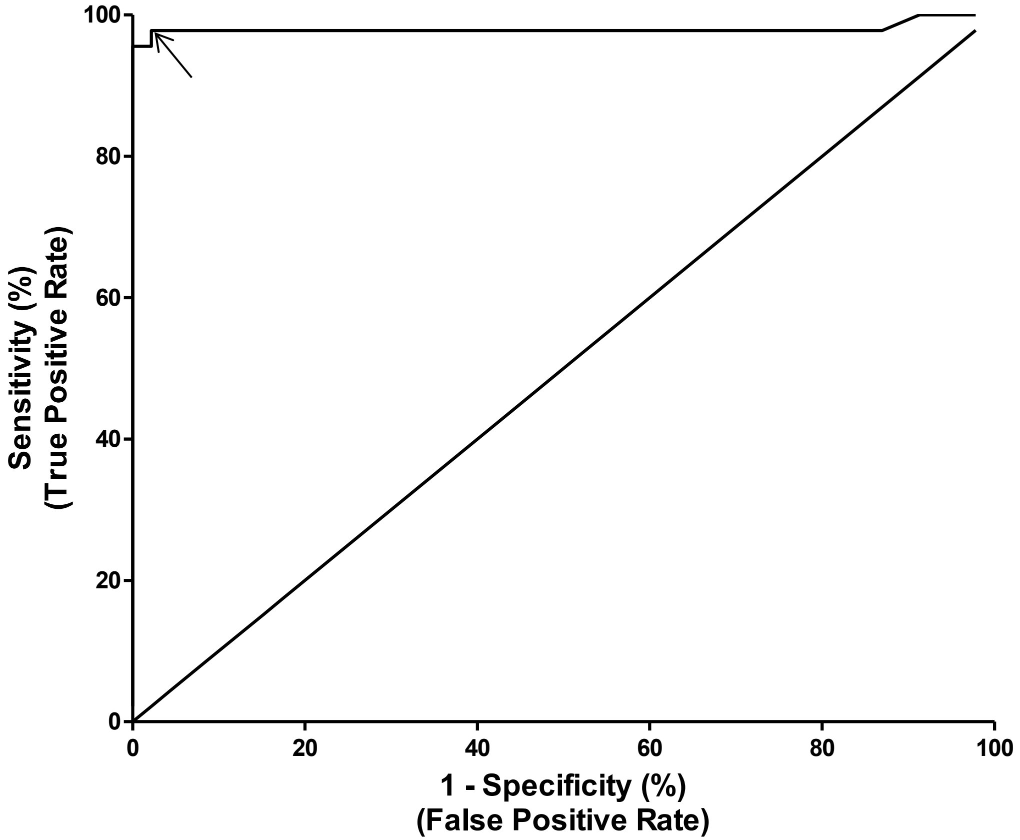

Arrow indicates a cutoff of 2.0

- AUC = 0.98 (0.94 – 1.02)
- P value <0.0001

Supplementary Figure 2. ROC curve analysis for EP-QuIC

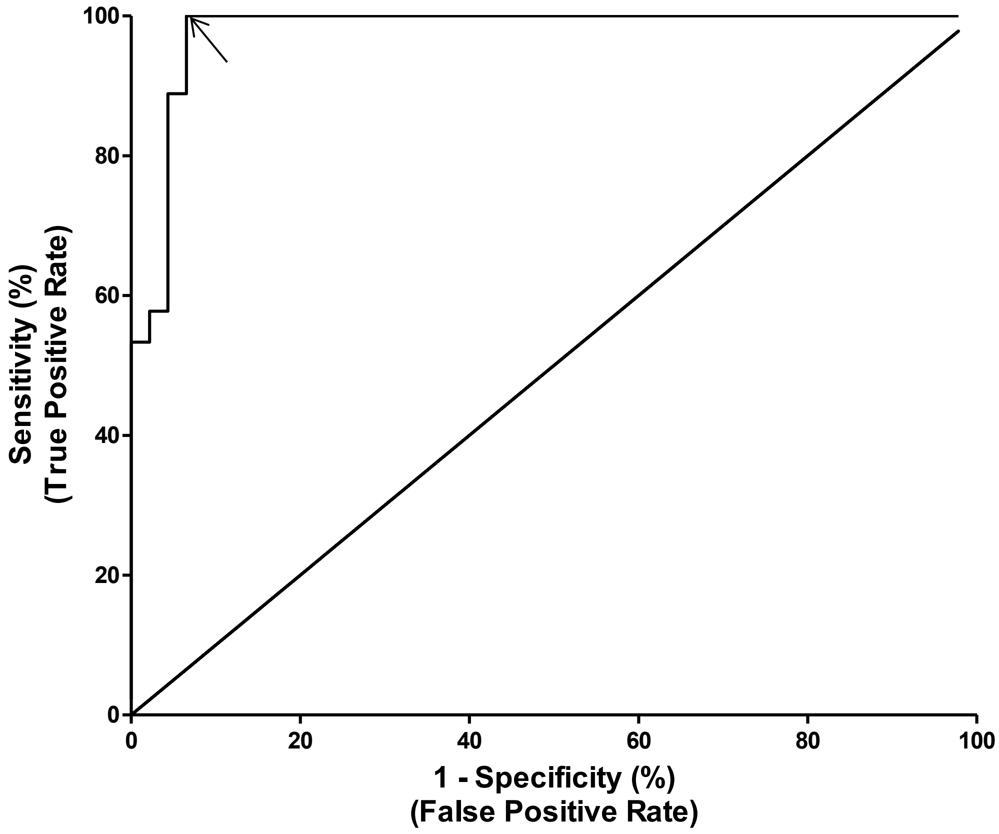

Arrow indicates a cutoff of 4.0

- AUC = 0.98 (0.95 – 1.01)
- P value <0.0001

Supplemental Table 1 Clinical Classification of Samples.

		Diagnosis	Number of cases
Breakdown of diagnoses:	CJD+	+ CJD	39
		+ CJD w/Alzheimers	4
		+ fCJD	2
	CJD-	Alzheimer's Disease	3
		Hydrocephalus	2
		Delirium	2
		Alzheimer's Disease / Dementia	1
		Probable Alzheimer's Disease	1
		Not CJD	1
		Dementia NOS	1
		Encephalitis	1
		Sub clinical Epilepsy	1
		Vascular Dementia	1
		Whipples Disease	1
		Transverse myelitis	1
		Status epilepticus with Hypothyroidism	1
		Lymphoma	1
		Probable Mitocondrial Disease	1
		MS	1
		Fronto-temporal dementia	1
		Myoclonus NYD	1
		Bipolar Disorder	1
		Septicemia	1
		Atypical brain stem stroke	1
		Psychosis	1
		aspiration pneumonia	1
		Hypoxic encephalopathy	1
		NYD	1
		Non Specific Dementia	1
		Chronic Hypoxia	1
		Coma rigidity, NYD	1
		Delusional-Psychiatric Disease NYD	1
		CVA	1
		HIV	1
		Parkinsons. Plus Syndrome	1
		Grade III Astrocytoma	1
		Not available	1
		Motor Neuro Disease	1
		Progressive cognitive decline	1
		Acute Glysomatosis	1
		Degenerative Brain Disorder, Unknown eitology	1
		Motorneuropathy	1
		Alcoholism	1
		Thiamine deficiency	1
		Paraneoplastic Syndrome	1

Supplementary Table 2. Clinical Diangoses of False positive, False negative and Indeterminate samples

	Diagnosis	# False Results		# Indeterminate	
		EP-QuIC	RT-QuIC	EP-QuIC	RT-QuIC
CJD+	(A) + CJD		1	1[a]	1[a]
CJD-	Alzheimer's Disease / Dementia			1	
	Probable Alzheimer's Disease			1	
	Status epilepticus with Hypothyroidism	1[b]			1[b]
	Acute Glysomatosis	1			

[a] same CSF sample

[b] same CSF sample

Supplementary Table 3. Inter-observer agreement analysis (Kappa test)*

A

		Test #1 Result		
		Positive	Negative	
Test #2 Result	Positive	**a**	b	m_1
	Negative	c	**d**	m_0
		n_1	n_0	n

(a) and (d) are the number of times that the two tests gave the same result. (b) and (c) are the number of times that the two tests did not agree.

$$\text{Kappa} = \frac{(p_o - p_e)}{(1 - p_e)}$$

Where the observed agreement $p_o = (a+d)/n$

and the expected agreement $p_e = [(n_1/n)*(m_1/n)] + [(n_0/n)*(m_0/n)]$

B

		RT-QuIC		
		Positive	Negative	
EP-QuIC	Positive	**43**	2	45
	Negative	0	**42**	42
		43	44	87

K = (0.98 – 0.50) / (1 – 0.50) = 0.95

* Indeterminate samples (two in RT-QuIC and three in EP-QuIC) were excluded from the analysis. A, Kappa calculation data layout; B, Kappa test for RT-QuIC and EP-QuIC reactions on 87 samples.
